# Association between pubertal development and elevated blood pressure in children

**DOI:** 10.1111/jch.14315

**Published:** 2021-07-03

**Authors:** Yanhui Li, Yanhui Dong, Zhiyong Zou, Di Gao, Xijie Wang, Zhaogeng Yang, Bin Dong, Jun Ma

**Affiliations:** ^1^ Institute of Child and Adolescent Health, School of Public Health Peking University Beijing China

**Keywords:** blood pressure, children and adolescents, hypertension, pubertal development, pubertal timing

## Abstract

Blood pressure (BP) increased with age and height development, but little was known about the effect of pubertal development on blood pressure in children. A cross‐sectional study was performed among 4146 children aged 7–12 years old in China. Pubertal development was assessed based on breast stages and testicular volume. The associations of pubertal development with BP levels and the rate of elevated blood pressure (EBP) were quantified using multiple linear and logistic regressions. We found that pubertal developmental level was positively correlated with BP, and children who experienced puberty onset and early pubertal timing had higher BP levels and prevalence of EBP. After adjusting for covariates, children experienced puberty onset had 3.84 and 2.24 mmHg increase in systolic blood pressure and diastolic blood pressure, and 70%, 53%, and 62% increased odds of EBP, ESBP, and EDBP, respectively, compared with those without puberty onset. Similar results were observed for children who had early pubertal timing. The change of BP in puberty is greater and the association between pubertal development and BP is stronger in girls than boys. These findings suggested that pubertal development could be an important independent factor and one critical period for the EBP progress. Monitoring and management of pubertal development are necessary particularly among girls.

## 1 INTRODUCTION

Elevated blood pressure (EBP) is an important risk factor for cardiovascular disease,^1^ and has been recognized as a big contributor to the global burden of disease and to global mortality.^2^ Hypertension rarely causes symptoms in the early stages, it is a silent killer, however, with the increase in prevalence of obesity and rapid urbanization, a higher prevalence of EBP in children and adolescents is observed.^3^ Childhood blood pressure (BP) is a strong predictor of adult BP,^4^, ^5^ and children with high‐increasing trajectory of BP from childhood to young adults had thicker carotid intima–media thickness and increased left ventricular mass index.^6^ It is known that BP increases with age, and BP increases rapidly during pubertal growth.^7^, ^8^ Puberty is a critical temporal window for the development of hypertension, and this period may be an important one to detect or prevent hypertension risk factors for adolescents’ future life.^1^

Previous studies found that pubertal developmental status is positively correlated with BP.^9^, ^10^ But the synchronization of aging, accelerated growth, and pubertal development has led to inconsistent results on whether the increase in BP during puberty is the role of pubertal development.^11^, ^12^ Recent studies have shown that there is a tendency towards earlier puberty onset and pubertal development.^13^, ^14^ Earlier puberty is associated with current physical and mental health and behaviors in children and adolescents, as well as adverse outcomes such as psychological disorders, cancer and cardiovascular diseases in adulthood.^15^, ^16^, ^17^ Taking the middle to late events as indicators of pubertal development, a lot of studies have found that earlier puberty is associated with increased BP.^16^, ^18^, ^19^ However, BP at late puberty is affected by many factors, and the association between late pubertal indicators and blood pressure reflected more than the effects of puberty onset and pubertal development. So studying the relationship between puberty onset‐related events and BP is crucial to explain the dramatic changes in adolescent BP. Nevertheless, the relationship between puberty onset and early pubertal timing and BP and the characteristics of BP at different stages of puberty are not clear.

The current study aims to describe the changes of BP in puberty and the effects of pubertal development, and analyze the association between pubertal development and EBP based on a cross‐sectional data among Chinese children, with a view of identifying high‐risk groups and providing evidence for early prevention and control of hypertension.

## 2 METHODS

### 2.1 Study population

We used the baseline information from a cohort of Chinese children recruited in 2017. Four schools at the Xiamen were selected with random cluster sampling. All children from two to six grades were included in each school.

**FIGURE 1:**
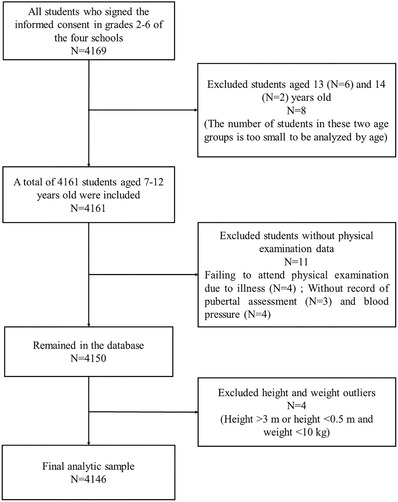
The selection process of participants

A total of 4161 children aged 7–12 years old were investigated at local schools in May 2017. We excluded 11 children with missing values on pubertal assessment and BP, and 4 children had abnormal records of height and weight. Finally, 4146 participants were enrolled in the study. The selection process of participants was shown in Figure 1. This study was approved by the Medical Ethical Committee of Peking University (Number: IRB00001052‐17026), and all the written informed consents were obtained from both parents of each child.

### 2.2 Physical examination

Anthropometric measurements including height, weight, and BP were collected and calibrated by trained staff using a standardized procedure. Height was measured to the nearest 0.1  cm using a portable stadiometer and weight was measured to the nearest 0.1  kg using a weight scale without shoes and in light clothing. Body mass index (BMI) was calculated as body weight (kg) divided by height (m) squared. Blood pressure was measured using an electronic sphygmomanometer (Omron HBP‐1100) that met the European Society of Hypertension International Protocol standard (revision 2010) and is recommend for clinical use.^20^ We used S cuff (18–22 cm) for 6–11 years old children and M cuff (22–32 cm) for 12 and above, with the inflatable bladder width covered ≥40% of the circumference of the arm, and the cuff bladder length covered ≥80%. The cuff was placed approximately 2 cm above the crease of the right arm elbow. All students seated comfortably at least 5‐min rest. Blood pressure including systolic blood pressure (SBP) and diastolic blood pressure (DBP) was measured two times with 1 min break between them. If the difference between the two measurements was larger than 10 mmHg, the third one was taken. The average value of the measurements was used for the analysis.

### 2.3 Pubertal assessment

Pubertal assessment was conducted separately for girls and boys. Breast development in girls was assessed by visual inspection and palpation based on the rating scales of Tanner. Testicular volume and genital development in boys were evaluated by comparative palpation and visual inspection with the Prader orchidometer and rating scales of Tanner. We classified participants into different pubertal stages (I–V) according to the development of secondary sexual characteristics^21^, ^22^, ^23^ (Table S1).

We defined pubertal stages I as prepuberty and others as puberty.^23^, ^24^ Children at early pubertal timing were those whose puberty onset age earlier than the first quartile among the children of the same sex.^25^ Pubertal developmental assessments for boys and girls were performed by two trained physicians or medical postgraduates of the same sex.

### 2.4 Elevated blood pressure definitions

We defined children with elevated systolic blood pressure (ESBP) or elevated diastolic blood pressure (EDBP) as elevated blood pressure (EBP) if their BP were equal or larger than the 90th percentiles of the referent age‐, sex‐, and height‐specific population (Chinese National BP Reference).^26^

### 2.5 Questionnaire investigation

The information on demographic characteristics and behaviors were collected by questionnaires. Children are required to report their own age, sex (boy or girl), date of birth and taste preference (partial salty, moderation, partial light, don't know) through questionnaires. Parents were asked to provide information on family history of hypertension (whether the father or mother had hypertension). Chronological age was calculated by subtracting the birth date from the examination date.

### 2.6 Statistical analysis

We presented mean values and standard deviations for continuous variables and numbers and percentages for categorical variables. Due to the small sample size in late puberty, we combined stage IV and stage V. We calculated the quartiles age of stage Ⅱ for boys and girls by using the probit regression analysis. Variance analysis and Chi‐square test were used to compare the differences between groups. Bonferroni method was used for multiple comparison. Multiple linear and logistic regression was conducted to analyze the relationship between pubertal development and BP levels and odds of EBP. We reported coefficient, odds ratio (OR) and 95% confidence intervals (CI) for the rough model and the adjusted model (adjusting for age, sex, BMI, taste preference, and family history of hypertension). Trend analysis was performed to explore the dose–response relationship between pubertal stages and BP levels and odds of EBP. We also performed a subgroup analysis stratified by sex. All data analysis was performed with SPSS 24.0, and result with a *p*‐value <.05 was considered statistically significant. GraphPad Prism 7 was used to create visual figures.

## 3 RESULTS

### 3.1 Characteristics of participants

Table 1 showed the characteristics of participants. A total of 4146 children, including 2391 boys and 1755 girls aged 7–12 years old, were included in this study. The prevalence of EBP, ESBP, and EDBP in the children was 22.5%, 18.6%, and 10.3%, respectively. There were 1846 children entering puberty, and the proportions of each Tanner stage were 55.5%, 20.8%, 14.3%, and 9.4%, respectively. The age, height, weight, BMI, SBP, DBP, and the prevalence of EBP, ESBP, EDBP increased with the Tanner stages.

**TABLE 1:** General characteristics of study participants

		Pubertal stage					
Characteristics	Total	I	II	III	IV‐V	*F/χ* ^2^	*p*
Number	4146	2300	863	594	389		
Age (year)	10.1 ± 1.5	9.2 ± 1.1	10.6 ± 1.2*	11.5 ± 0.9*	11.8 ± 0.7*	1345.94	<.01
Girls (%)	1755(42.3%)	609(26.5%)	477(55.3%)*	360(60.6%)*	309(79.4%)*	596.62	<.01
Height (cm)	142.9 ± 11.1	136.6 ± 8.0	145.9 ± 8.6*	153.7 ± 7.6*	157.3 ± 6.8*	1304.08	<.01
Weight (kg)	36.4 ± 10.8	31.7 ± 8.5	38.3 ± 9.8*	43.4 ± 9.8*	48.8 ± 9.1*	580.49	<.01
BMI (kg/m^2^)	17.5 ± 3.3	16.8 ± 3.1	17.8 ± 3.3*	18.3 ± 3.1*	19.6 ± 3.1*	118.27	<.01
SBP (mmHg)	105.7 ± 11.5	102.5 ± 10.8	107.1 ± 10.7*	110.8 ± 10.8*	113.6 ± 10.8*	187.55	<.01
DBP (mmHg)	63.5 ± 9.4	61.8 ± 9.0	64.6 ± 9.0*	65.6 ± 10.2*	67.4 ± 9.3*	63.65	<.01
ESBP (%)	773(18.6%)	343(14.9%)	166(19.2%)*	142(23.9%)*	122(31.4%)*	73.63	<.01
EDBP (%)	427(10.3%)	184(8.0%)	98(11.4%)*	85(14.3%)*	60(15.4%)*	35.61	<.01
EBP (%)	933(22.5%)	412(17.9%)	208(24.1%)*	172(29.0%)*	141(36.2%)*	85.37	<.01
Family history of hypertension	450(10.9%)	239(10.4%)	105(12.2%)	61(10.3%)	45(11.6%)	2.46	.48
Taste preference						54.00	<.01
Partial salty	812(20.1%)	485(21.6%)	171(20.3%)	99(17.0%)	57(15.0%)*		
Moderation	2482(61.4%)	1280(57.1%)	535(63.4%)*	400(68.8%)*	267(70.4%)*		
Partial light	509(12.6%)	309(13.8%)	98(11.6%)	58(10.0%)	44(11.6%)		
Don't know	242(6.0%)	167(7.5%)	40(4.7%)*	24(4.1%)*	11(2.9%)*		

Continuous variables were expressed by mean values ± standard deviations, and categorical variables were expressed by numbers and percentages.

Abbreviations: BMI, body mass index; DBP, diastolic blood pressure; EBP, elevated blood pressure; EDBP, elevated diastolic blood pressure; ESBP, elevated systolic blood pressure; SBP, systolic blood pressure.

* Indicated that the differences of characteristics were statistically significant compared with the pubertal stage I (*p *< .05).

### 3.2 Puberty and blood pressure

**FIGURE 2:**
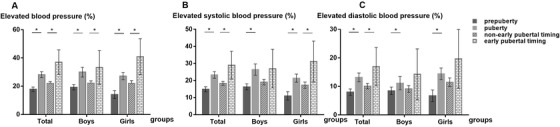
The prevalence of elevated blood pressure at different pubertal developmental status in Chinese children aged 7–12 years. * *p *< .05

The levels of SBP and DBP at different pubertal developmental status were presented in Table 2. Children who had puberty onset had higher SBP and DBP levels compared with those who had not (*p *< .05). In each age group, higher BP levels were observed in participants who experienced puberty onset than those peers in prepuberty. The P_25_ ages of stage II for boys and girls were 10.4 and 8.6 years, respectively. There were 3% of students experiencing early pubertal timing. The levels of SBP and DBP in the early pubertal timing group were significantly higher than that in the non‐early group (*p *< .05). Consistent with BP levels, the prevalence of EBP, ESBP, and EDBP was distinctly higher in the puberty group and early pubertal timing group (Figure 2).

**TABLE 2:** The levels of BP at different pubertal developmental status in Chinese children aged 7–12 years

Age	Group	N	SBP (mmHg)	Boys’ SBP (mmHg)	Girls’ SBP (mmHg)	DBP (mmHg)	Boys’ DBP (mmHg)	Girls’ DBP (mmHg)
7	Prepuberty	318	100.40(99.22,101.58)	102.61(101.01,104.22)	97.52(95.89,99.14)	60.37(59.35,61.39)	61.22(59.83,62.61)	59.26(57.78,60.75)
	Puberty	17	106.35(102.05,110.66)^*^	–	106.35(102.05,110.66)^*^	65.08(60.47,69.69)^*^	–	65.08(60.47,69.69)^*^
8	Prepuberty	826	101.18(100.42,101.94)	103.17(102.23,104.10)	97.55(96.34,98.75)	61.30(60.67,61.93)	61.96(61.14,62.77)	60.10(59.16,61.04)
	Puberty	94	106.98(104.71,109.24)^*^	110.29(99.82,120.76)	106.67(104.32,109.02)^*^	65.57(63.36,67.78)^*^	67.21(57.94,76.48)	65.41(63.10,67.73)^*^
9	Prepuberty	636	103.77(102.96,104.58)	104.58(103.67,105.48)	100.46(98.72,102.19)	62.77(62.11,63.42)	63.15(62.42,63.88)	61.21(59.75,62.67)
	Puberty	205	106.25(104.83,107.67)^*^	107.51(103.80,111.22)	106.06(104.52,107.61)^*^	64.87(63.70,66.03)^*^	62.68(58.36,67.00)	65.18(63.99,66.38)^*^
10	Prepuberty	364	104.06(102.96,105.16)	104.76(103.63,105.89)	98.54(94.82,102.27)	62.54(61.67,63.41)	62.76(61.83,63.70)	60.78(58.50,63.06)
	Puberty	410	107.53(106.41,108.65)^*^	110.02(107.92,112.11)^*^	106.57(105.25,107.89)^*^	65.64(64.73,66.55)^*^	65.68(63.78,67.59)^*^	65.62(64.58,66.66)^*^
11	Prepuberty	135	104.46(102.72,106.20)	105.28(103.50,107.07)	95.18(89.99,100.38)	62.05(60.28,63.82)	62.19(60.29,64.09)	60.45(55.99,64.91)
	Puberty	604	110.38(109.54,111.23)^*^	110.42(109.13,111.71)^*^	110.36(109.25,111.46)^*^	65.70(64.94,66.46)^*^	64.10(62.92,65.28)	67.11(66.16,68.06)^*^
12	Prepuberty	21	105.08(100.03,110.13)	104.61(99.07,110.16)	109.50(52.32,166.68)	60.44(55.79,65.10)	59.12(54.39,63.86)	73.00(47.59,98.41)
	Puberty	516	112.44(111.51,113.37)^*^	113.24(111.91,114.56)^*^	111.57(110.27,112.87)	65.44(64.59,66.29)^*^	63.87(62.61,65.13)	67.15(66.05,68.25)
Total	Prepuberty	2300	102.47(102.03,102.91)	104.01(103.50,104.52)	98.20(97.39,99.02)	61.81(61.44,62.18)	62.38(61.94,62.81)	60.23(59.58,60.89)
	Puberty	1846	109.65(109.15,110.16)^*^	111.33(110.50,112.15)^*^	108.63(108.00,109.26)^*^	65.51(65.07,65.94)^*^	64.25(63.49,65.02)^*^	66.28(65.76,66.79)^*^
Pubertal timing	Non‐early	4022	105.57(105.21,105.92)	106.04(105.58,106.49)	104.92(104.36,105.48)	63.41(63.12,63.69)	62.87(62.48,63.25)	64.15(63.71,64.58)
	Early	124	109.03(107.11,110.95)^*^	110.46(107.67,113.24)^*^	107.56(104.89,110.23)	65.12(63.18,67.06)^*^	65.14(62.32,67.95)	65.10(62.35,67.85)

This table shows the mean and 95% CI of BP levels.

Abbreviations: DBP, diastolic blood pressure; SBP, systolic blood pressure.

* Indicated that the differences in blood pressure levels were statistically significant between the prepuberty and puberty groups or pubertal timing early group and non‐early groups (*p *< .05).

– indicates there is no data (because the number of boys entering puberty is zero in the 7‐year‐old group).

Stratification by sex, both boys and girls who had puberty onset had higher SBP and DBP levels, but the difference of SBP and DBP between the puberty group and prepuberty group was greater in girls than that in boys (10.4 and 6.0 mmHg vs. 7.3 and 1.9 mmHg). Girls with early pubertal timing had a higher prevalence of ESBP, not boys.

### 3.3 Association between pubertal development and elevated BP

Pubertal development was related with higher BP levels (Figure S1). After adjusting for age, height, weight, and other covariates, a significant positive correlation still existed. Puberty onset was associated with 3.84 (95% CI: 2.92–4.75) mmHg and 2.24 (95% CI: 1.39–3.09) mmHg increased in SBP and DBP (Table 3). Children with early pubertal timing had an increased SBP levels by average 3.19 (95% CI: 1.37–5.01) mmHg compared to those with non‐early pubertal timing. A significant increase in SBP and DBP levels across the pubertal stages was also observed (*p*_trend_ < .01).

**TABLE 3:** The relationship between different pubertal developmental status and BP levels in Chinese children aged 7–12 years

Group		N	SBP (mmHg)	Boys’ SBP (mmHg)	Girls’ SBP (mmHg)	DBP (mmHg)	Boys’ DBP (mmHg)	Girls’ DBP (mmHg)
Puberty onset	Prepuberty	2300	0 (reference)	0 (reference)	0 (reference)	0 (reference)	0 (reference)	0 (reference)
	Puberty	1846	3.84(2.92,4.75)^*^	3.65(2.41,4.88)^*^	4.11(2.71,5.52)^*^	2.24(1.39,3.09)^*^	1.15(‐0.04,2.33)	3.39(2.18,4.61)^*^
Pubertal timing	Non‐early	4022	0 (reference)	0 (reference)	0 (reference)	0 (reference)	0 (reference)	0 (reference)
	Early	124	3.19(1.37,5.01)^*^	2.91(0.41,5.41)^*^	4.27(1.56,6.99)^*^	1.54(‐0.14,3.22)	1.25(‐1.15,3.64)	2.61(0.26,4.96)^*^
Pubertal stage	Stage I	2300	0 (reference)	0 (reference)	0 (reference)	0 (reference)	0 (reference)	0 (reference)
	Stage II	863	3.09(2.14,4.04)^*^	2.39(1.08,3.70)^*^	3.80(2.37,5.22)^*^	2.05(1.17,2.93)^*^	1.36(0.09,2.63)^*^	3.10(1.87,4.34)^*^
	Stage III	594	6.02(4.80,7.24)^*^	5.53(3.83,7.23)^*^	6.13(4.27,7.99)^*^	2.74(1.61,3.88)^*^	0.07(‐1.58,1.72)	4.64(3.04,6.25)^*^
	Stage IV‐V	389	7.22(5.72,8.71)^*^	9.70(7.19,12.21)^*^	6.11(3.86,8.35)^*^	3.75(2.36,5.14) ^*^	3.04(0.60,5.47)^*^	3.75(1.81,5.70)^*^

This table shows the coefficient and 95%CI of multiple linear regression.

Abbreviations: DBP, diastolic blood pressure; SBP, systolic blood pressure.

* Indicated that the differences in blood pressure levels were statistically significant compared with the reference (*p *< .05).

Adjusting for children's age, (sex), body mass index, taste preference, and family history of hypertension.

As shown in logistics regression (Figure S2), there was also a strong positive correlation between pubertal development and odds of EBP. Children who had puberty onset were 70% (OR = 1.70, 95% CI = 1.34–2.14), 53% (OR = 1.53, 95% CI = 1.19–1.96), and 62% (OR = 1.62, 95% CI = 1.19–2.21) higher odds of EBP, ESBP, and EDBP than prepubertal children. The odds of EBP in children with early pubertal timing was also increased by 64% (OR = 1.64, 95% CI = 1.10–2.45) compared with those whose pubertal timing was non‐early (Table 4). Children undergoing higher pubertal stages had a tendency for increased odds of EBP, ESBP, and EDBP (*p*_trend_ < .01).

**TABLE 4:** The association between different pubertal developmental status and EBP in Chinese children aged 7–12 years

	Group	EBP	Boys’ EBP	Girls’ EBP	ESBP	Boys’ ESBP	Girls’ ESBP	EDBP	Boys’ EDBP	Girls’ EDBP
Puberty onset	Prepuberty	1 (reference)	1 (reference)	1 (reference)	1 (reference)	1 (reference)	1 (reference)	1 (reference)	1 (reference)	1 (reference)
	Puberty	1.70(1.34,2.14)^*^	1.73(1.27,2.37)^*^	1.69(1.17,2.44)^*^	1.53(1.19,1.96)^*^	1.66(1.19,2.30)^*^	1.42(0.95,2.13)	1.62(1.19,2.21)^*^	1.36(0.88,2.12)	2.08(1.30,3.33)^*^
Pubertal timing	Non‐early	1 (reference)	1 (reference)	1 (reference)	1 (reference)	1 (reference)	1 (reference)	1 (reference)	1 (reference)	1 (reference)
	Early	1.64(1.10,2.45)^*^	1.41(0.80,2.50)	1.97(1.09,3.54)^*^	1.52(0.99,2.32)	1.34(0.74,2.44)	1.85(0.98,3.48)	1.35(0.80,2.28)	1.18(0.53,2.64)	1.60(0.78,3.28)
Pubertal stage	Stage I	1 (reference)	1 (reference)	1 (reference)	1 (reference)	1 (reference)	1 (reference)	1 (reference)	1 (reference)	1 (reference)
	Stage II	1.57(1.23,2.01)^*^	1.46(1.04,2.06)^*^	1.65(1.13,2.40)^*^	1.41(1.08,1.84)^*^	1.37(0.95,1.96)	1.38(0.91,2.10)	1.52(1.10,2.10)^*^	1.31(0.82,2.09)	1.91(1.17,3.11)^*^
	Stage III	2.32(1.70,3.17)^*^	2.34(1.52,3.61)^*^	2.23(1.38,3.59)^*^	2.09(1.50,2.92)^*^	2.27(1.44,3.57)^*^	1.76(1.04,2.96)^*^	2.15(1.42,3.25)^*^	1.41(0.76,2.61)	3.07(1.67,5.63)^*^
	Stage IV‐V	2.82(1.94,4.10)^*^	4.21(2.34,7.58)^*^	2.39(1.36,4.21)^*^	2.65(1.79,3.94)^*^	5.04(2.76,9.20)^*^	1.83(0.99,3.38)	2.10(1.27,3.46)^*^	2.10(0.92,4.80)	2.43(1.17,5.05)^*^

This table shows the OR and 95%CI of multiple logistics regression.

Abbreviations: EBP, elevated blood pressure; EDBP, elevated diastolic blood pressure; ESBP, elevated systolic blood pressure.

* Indicated that the odds of elevated blood pressure were statistically significant compared with the reference (*p *< .05).

Adjusting for children's age, (sex), body mass index, taste preference, and family history of hypertension.

The similar results were found in boys and girls, but the association of puberty onset and early pubertal timing on BP levels and odds of EBP was even stronger among girls than in boys.

## 4 DISCUSSION

To our knowledge, this is the first study to date to analyze the associations between pubertal development and BP in Chinese children. In this study, we found that the pubertal development in children was positively correlated with BP levels and EBP. In addition, the puberty could be an independent influencing factor and a potential critical period for the development of EBP. Children who had puberty onset or experienced early pubertal timing had higher BP levels and odds of EBP. We also found that girls had larger increase in BP levels during puberty, and had a stronger association between pubertal development and BP levels and EBP. More attention and health education should be paid to puberty of children to reduce the potential risks of chronic diseases in advance.

A birth cohort study in Hong Kong indicated that the age of puberty onset was negatively correlated with BP levels.^10^ Another cohort study assessing secondary sexual development by visual inspection in Afro‐Caribbean children also found that breast development and testicular volume was positively correlated with SBP.^9^ These evidences were in line with our findings. On the contrary, in a small sample of black girls in a low socioeconomic area, higher breast development stage was related with higher BP, but it did not explain a significant proportion of variability in BP.^27^ Another study also found puberty stage was correlated to SBP and DBP, but it was not included in the regression model.^28^ Several researches speculated that the effect of sexual maturation on BP appeared to operate through age, height, and body size.^11^, ^12^ In this study, we investigated the differences in BP levels between puberty group and prepuberty group by age group, and controlled the effects of age, and BMI in regression analysis. It was found that puberty onset was an independent influencing factor of BP, and pubertal development was closely related to elevated BP. The inconsistency of current findings may be attributable to the racial difference, sample size, age range of participants, and the various evaluations of pubertal development.^29^

The mechanisms linking pubertal development and BP are not fully understood, but some researches may suggest some underlying potential mechanisms. Sex hormones have been found to be involved in the regulation of BP levels. Estrogen could stimulate the release of endothelium‐derived vasodilator factors, inhibit the renin‐angiotensin system, and be related with decreased BP. While testosterone can raise BP by stimulating the renin–angiotensin–aldosterone system.^30^, ^31^ But how sex hormones regulate BP during puberty has not been revealed. Preliminary researches found that the significant increase in dihydrotestosterone, testosterone, estrone, and estradiol with pubertal development.^32^ Therefore, the effects of pubertal development on BP may be mediated by sex hormones. Cohort studies found that children who experienced early pubertal maturation had lower subsequent physical activity,^33^ which was inversely associated with blood pressure.^34^ Pubertal development may also influence blood pressure levels by regulating physical activity. In addition, study had shown that cardiovascular autonomic function plays an important role in increasing BP levels associated with increased modulation of vagal tone of the heart in puberty but does not in the preadolescent.^35^

The sex differences in pubertal development and BP also in part help to validate the potential mechanisms by sex hormones. It is well known that boys have higher prevalence of hypertension than girls.^36^ Unexpectedly, we detected that girls’ BP growing larger during puberty. This may be attributed to the earlier maturation in girls compared with boys. Some studies showed that the association between pubertal development and early pubertal timing and high BP exists only in girls.^9^, ^37^ We discovered that puberty onset increased SBP and DBP in boys and girls, but after adjusting for covariates, the relationship between pubertal development and BP levels and EBP was stronger in girls than boys. Some studies pointed out that the effect of pubertal development on BP appeared to operate through body fat.^12^, ^28^ Prior studies showed that adolescent girls gained more body fat than boys.^38^ More researches are needed in the future to confirm this result and elucidate the possible mechanisms.

Based on a relatively large sample size, this study assessed pubertal developmental status using clinical examinations, which generally were considered the gold standard. The application of visual inspection and palpation to assess breast development was helpful to identify adipose tissue and true breast tissue, and to reduce misclassification bias. We found that pubertal development was related with higher blood pressure levels, which reminds us that puberty could be a vital window for the development of BP, possibly setting the stage for future BP levels. We should pay more attention to children and advocate healthy eating and exercise habits to prevent BP elevation. In the past, girls may be neglected, and the supervision and prevention of hypertension in this group should be strengthened in the future.

There are several limitations in this study. Firstly, this study is a cross‐sectional study, which limits the judgment of causality. However, this study provides evidence to explore the relationship between puberty onset and pubertal development and blood pressure. Secondly, the small number of stages IV and V prevents us from covering the entire puberty, and fail to observe the characteristics of BP changes during complete adolescence. Thirdly, the US pediatric BP guidelines recommended that hypertension should be defined at least three occasions’ examination in different time points in children, we only had one BP measurement with two readings in a single visit, which may overestimate BP levels. Fourthly, there are different criteria for the determination of EBP in children and adolescents, including the screening criteria of American Academy of Pediatrics, the Chinese National BP Reference and the Simplified Pediatric Hypertension Criteria.^26^, ^36^, ^39^ We chose the Chinese National BP Reference suitable for Chinese children and adolescents. The effect values of the association between pubertal development and EBP may be different according to different criteria, but it is unlikely to affect the positive association between them. Moreover, the consistent results were found for the association between pubertal development and BP levels and EBP.

## 5 CONCLUSIONS

In this large population study of children, we found that puberty could be an influencing factor of EBP independent of age and BMI. Pubertal development and early pubertal timing were positively correlated with increased BP levels and EBP, suggesting that puberty could be a critical period for the development of BP, and early monitoring of BP in childhood should be strengthened. The early identification and appropriate management in children, especially girls who were undergoing puberty onset and early pubertal timing, is important to prevent the development of hypertension and its related cardiovascular diseases in later life.

## CONFLICT OF INTEREST

The authors have no relevant financial or non‐financial interests to disclose.

## AUTHOR CONTRIBUTIONS

Yanhui Li designed the study, carried out the data collection and analysis, drafted the initial manuscript, and reviewed and revised the manuscript. Yanhui Dong designed the study, carried out the data collection, drafted the initial manuscript, and reviewed and revised the manuscript. Zhiyong Zou designed the study, critically reviewed the manuscript and contributed to the interpretation of results. Di Gao, Xijie Wang, Zhaogeng Yang, and Bin Dong carried out the data collection and initial data analysis, drafted the initial manuscript, and reviewed and revised the manuscript. Jun Ma conceptualized and designed the study, coordinated and supervised data collection, and critically reviewed the manuscript for important intellectual content. All authors approved the final manuscript as submitted and agree to be accountable for all aspects of the work.

## Supporting information

Supplementary information
