# Supplementary material for: Association between pubertal development and elevated blood pressure in children
Source: J Clin Hypertens (Greenwich). 2021 Jul 3;23(8):1498–505. doi: 10.1111/jch.14315 (PMC8678653; doi:10.1111/jch.14315)
Supplement: Supplementary file 1 — Supplementary information [file JCH-23-1498-s001.docx]

Supplementary Table 1 Tanner staging criteria for secondary sexual characteristics

|  | Pubertal stages | | | | |
| --- | --- | --- | --- | --- | --- |
|  | Stage Ⅰ | Stage Ⅱ | Stage Ⅲ | Stage Ⅳ | Stage Ⅴ |
| Girls | Undeveloped, flat breasts. | Elevation of breast and papilla as a small mound, the glands may have a slight tenderness, enlargement of areola diameter. | Further enlargement of breast and areola, with no separation of their contours. The papilla begins to swell and protrude from the skin, and the areola begins to stain | Projection of areola and papilla to form a secondary mound above the level of the breast. | Projection of papilla only, due to recession of the areola to the general contour of the breast. And breast develops into an adult state. |
| Boys | Testes, scrotum, and penis are of about the same size and proportion as in early childhood. | The scrotum and testes have enlarged (≥4ml) and there is a change in the texture of the scrotal skin. There is also some reddening of the scrotal skin. | Growth of the penis has occurred, at first mainly in length but with some increase in breadth. There has been further growth of testes and scrotum. | Penis further enlarged in length and breadth with development of glans. Testes and scrotum further enlarged. There is also further darkening of the scrotal skin. | Genitalia adult in size and shape. |


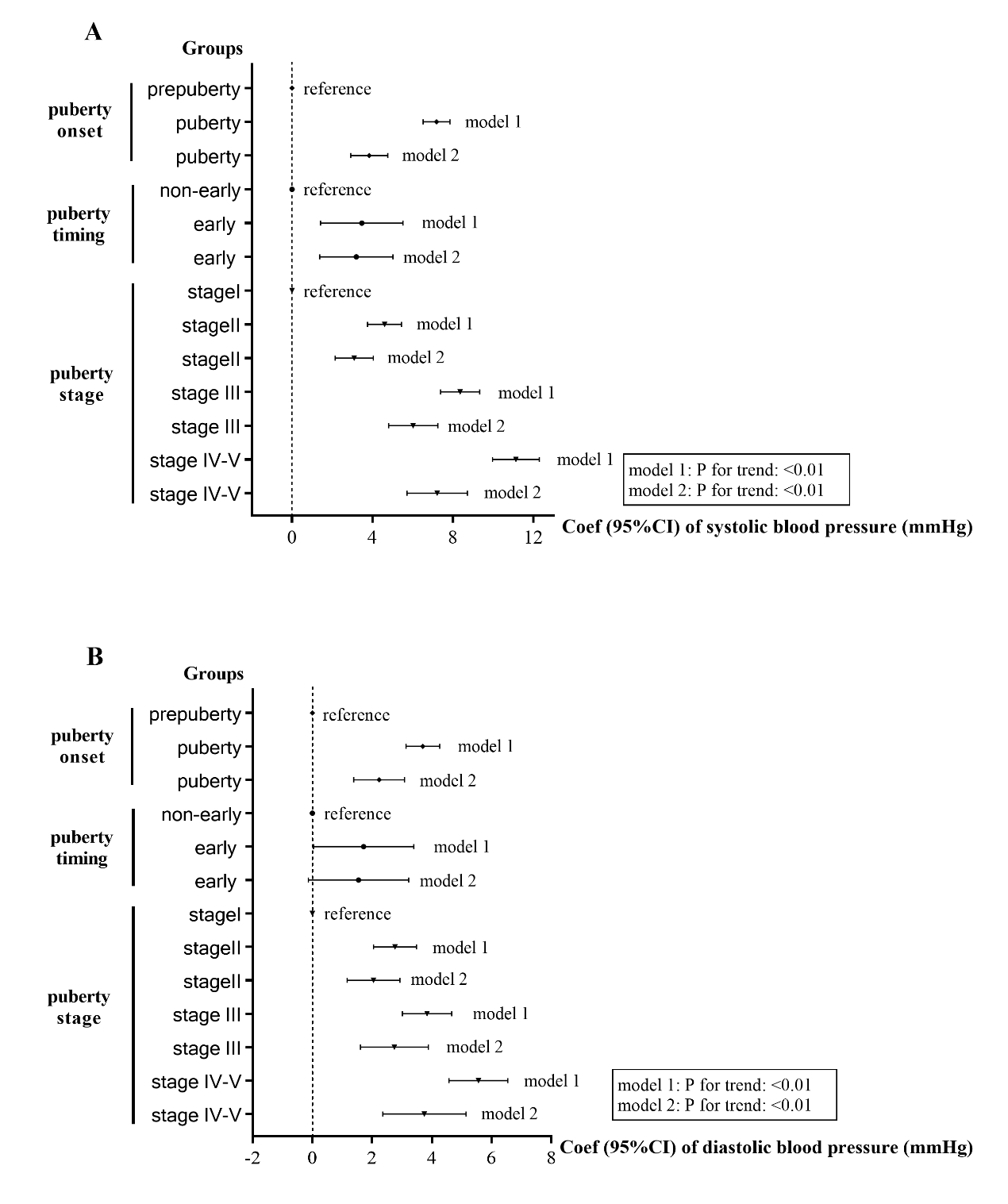
Supplementary Figure 1 The association between pubertal developmental status and blood pressure levels in different models

Model 1 did not adjust any factors.

Model 2 adjusted for children’s age, sex, body mass index, taste preference and family history of hypertension.


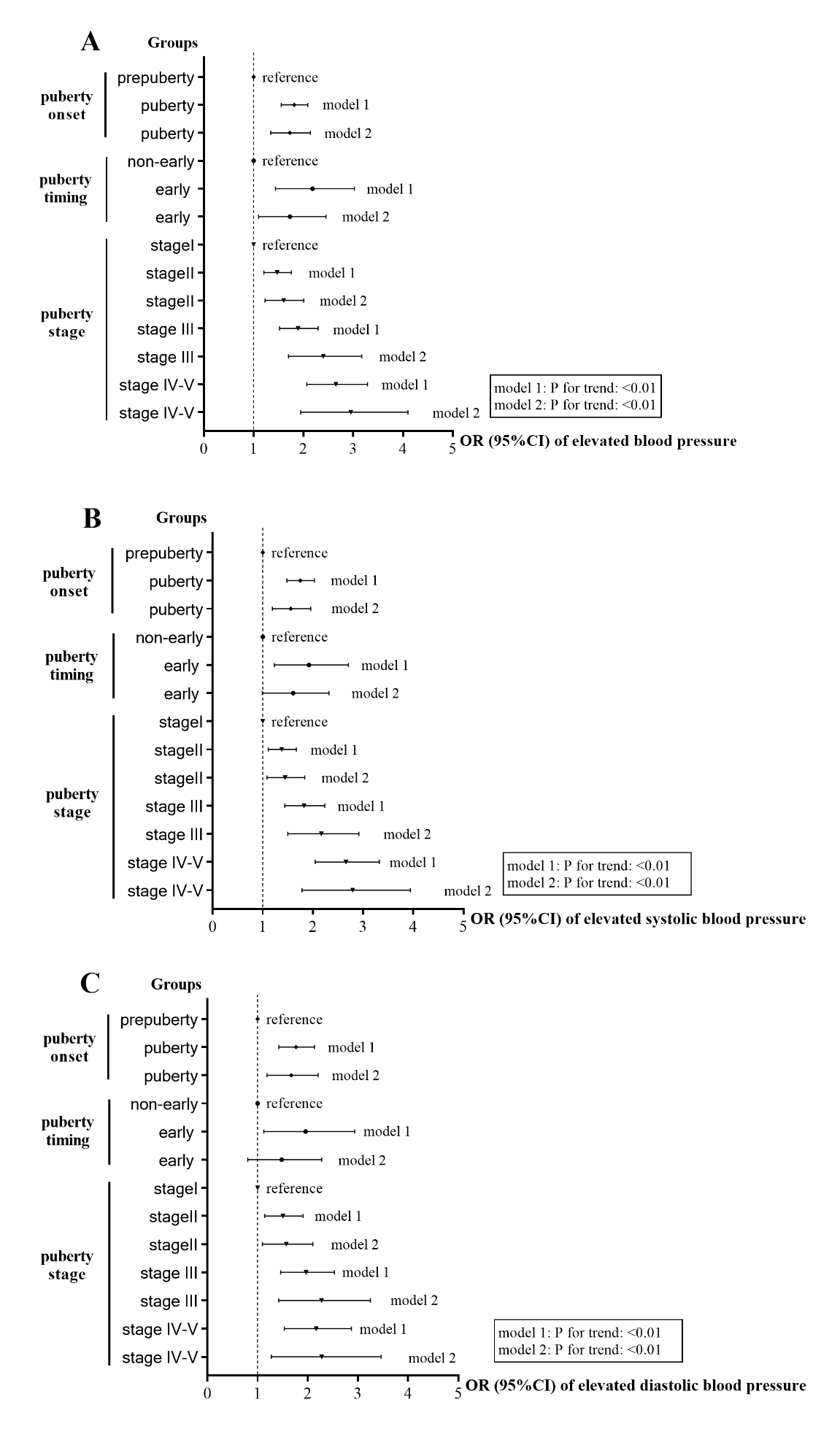
Supplementary Figure 2 The association between pubertal developmental status and odds ratio of elevated blood pressure in different models

Model 1 did not adjust any factors

Model 2 adjusted for children’s age, sex, body mass index, taste preference and family history of hypertension.
